# Bi-component modeling of cerebrospinal fluid outflow using Time-SLIP MRI

**DOI:** 10.1186/s12987-026-00791-9

**Published:** 2026-03-12

**Authors:** Vadim Malis, Mitsue Miyazaki

**Affiliations:** Department of Radiology, University of California-San Diego, 9427 Health Sciences Drive, La Jolla, CA 92093-0997 USA

**Keywords:** CSF outflow, Glymphatic, Time-SLIP

## Abstract

**Background:**

Understanding egress outflow of cerebrospinal fluid (CSF) is essential for exploring its role in brain health and its relationship to neurodegenerative diseases. Existing models often fail to capture the complex flow dynamics, particularly distinguishing rapid bulk flow from slower, perfusion-like components. This study aims to develop and validate a bi-component model that characterizes these distinct signal patterns using Time-Spatial Labeling Inversion Pulse (Time-SLIP) MRI data.

**Methods:**

A bi-component model is proposed by combining a Gaussian function (to represent fast bulk outflow) with a Г-variate function (representing slower, perfusion-like flow). Validation phantom studies were conducted using a sealed water phantom with computer-controlled bulk flow. In-vivo meninges images in eight healthy subjects were acquired on a clinical 3T MRI using Time-SLIP with 3D single-shot FSE. Signal Increase Ratio (SIR) from these experiments were fitted using the proposed bi-component model with fitting accuracy assessed using coefficient of determination (R^2^), Sum of Squared estimate of Errors (SSE), and Root Mean Square Error (RMSE).

**Results:**

Phantom studies showed superior fitting of bulk fluid dynamics by the Gaussian component, confirmed by lower SSE, RMSE and higher R^2^. The in-vivo results demonstrated that the bi-component model provided a superior fit, capturing two distinct components of CSF outflow, which are hypothesized to correspond to fast bulk flow and a slower, perfusion-like component, which could be a mixture of CSF and interstitial fluid.

**Conclusion:**

The bi-component model enhances characterization of CSF egress outflow from intrinsic labeling by Time-SLIP, providing potential quantitative biomarkers for the assessment of neurodegenerative diseases.

**Supplementary information:**

The online version contains supplementary material available at 10.1186/s12987-026-00791-9.

## Background

Effective clearance of metabolic waste from the brain is essential for maintaining brain health and preventing neurological diseases. A growing body of research indicates that the brain’s waste clearance is largely mediated by cerebrospinal fluid (CSF) flow through the glymphatic or neurofluid system, where it exchanges with interstitial fluid (ISF) in the brain parenchyma to flush out soluble metabolites, which then drain along perivascular space and egress pathways [1]. Impairment of these CSF outflow pathways has been linked to aging [2] and may contribute to the elevated risk of Alzheimer’s disease and other dementias in the elderly [3, 4]. Imaging the pathways and rate of CSF outflow in humans has historically been challenging, with techniques such as computed tomography (CT) myelography and radionuclide cisternography requiring invasive intrathecal tracer injections to visualize CSF dynamics and localize leaks [5, 6]. Two-photon microscopy, although invasive and confined to animal models, has enabled high-resolution visualization of paravascular CSF influx and CSF–ISF exchange in rodent brains, providing mechanistic insights into the glymphatic system [7].

In recent years, MRI-based techniques have gained increasing traction for investigating CSF dynamics *in-vivo* in human brain [8]. Contrast-enhanced MRI with intrathecal gadolinium tracers have visualized meningeal lymphatic vessels and confirmed CSF egress via dural lymphatics to cervical lymph nodes and via arachnoid granulations into venous sinuses, but these methods require invasive administration and exhibit very slow clearance kinetics (tens of hours) [9]. Noninvasive MRI methods have also been explored. Phase-contrast (PC) MRI has been applied to measure pulsatile CSF flow in central channels (such as aqueduct stroke volume in hydrocephalus) by encoding velocity oscillations with the cardiac cycle [10, 11]. While phase-contrast MRI is excellent for capturing fast oscillatory flow, it is less suited for quantifying slow perfusion-like components. Diffusion-weighted MRI has been proposed to assess glymphatic function, but it provides only indirect surrogate metrics rather than a direct visualization of CSF movement [12, 13]. Another non-contrast MRI technique allowing for visualization of CSF flow is Time-Spatial Labeling Inversion Pulse (Time-SLIP) MRI, which uses combination of spatially selective and non-selective inversion pulses to tag CSF and trace its flow dynamics [14, 15]. Yamada et al. also demonstrated the difference in CSF movement visualization between PC and Time-SLIP [16]. Recent brain studies with Time-SLIP MRI have demonstrated that intrinsic CSF outflow declines significantly with age in healthy adults, reflecting impaired glymphatic clearance [17]. Another study showed that a three-week aerobic exercise regimen in previously sedentary individuals enhanced CSF clearance, predominantly via the inferior parasagittal dura route to the superior sagittal sinus (SSS) [18]. While these initial studies demonstrate ability to visualize CSF outflow patterns at complexed meninges structure including SSS and parasagittal dura (PSD), interpreting this dynamic signals remains challenging.

A number of models describing glymphatic transport have been proposed, including diffusion-based clearance [19], pressure-driven bulk flow [20], dispersion combining flow and diffusion [21], two-compartment kinetics [22], and optimal mass-transport mapping [23]. Tracer experiments provide direct evidence of distinct clearance phases. Cserr et al. found that a large 2000 kDa dextran tracer injected into brain tissue was cleared rapidly, on the order of minutes, presumably via bulk flow along perivascular routes, whereas a smaller ~40 kDa tracer (horseradish peroxidase) dispersed more and exhibited a more prolonged washout [24]. Consistently, in-vivo two-photon imaging showed an initial fast distribution of CSF tracers (e.g. 3 kDa and 40 kDa dextrans) along arterial perivascular spaces, followed by slower clearance from the tissue, with the largest dextrans largely confined to perivascular channels [1, 25]. Dynamic contrast-enhanced MRI in rats also demonstrates a pattern with an early rapid influx of intrathecal tracer into the brain, followed by a slower elimination phase [22]. In humans, MRI studies with intrathecal gadobutrol demonstrated clearance kinetics with multiple peaks [26].

For Time-SLIP data analysis continuous analytical function like the Г-variate function has been proposed [17, 18], however, it may not accurately reflect the full complexity of CSF outflow mechanism due to the potential presence of multiple components. To address this gap, we propose a bi-component model comprising a Gaussian function for the fast bulk flow component and a Г-variate function for the slower, perfusion-like component. Both functions are analytic and continuous, allowing separate quantification of rapid and slow outflow phases.

In this work, we first use a controlled phantom study to validate the Gaussian function as an accurate model for fast bulk flow. We then apply the full bi-component model to preliminary in-vivo Time-SLIP data from healthy volunteers, demonstrating enhanced fitting accuracy and a physiologically meaningful separation between rapid bulk and slower perfusion-like CSF outflow dynamics. This framework aims to establish a foundation for future studies of age- and disease-related alterations in CSF clearance pathways.

## Methods

### Time-spatial labeling inversion pulse (Time-SLIP)

**Fig. 1:**
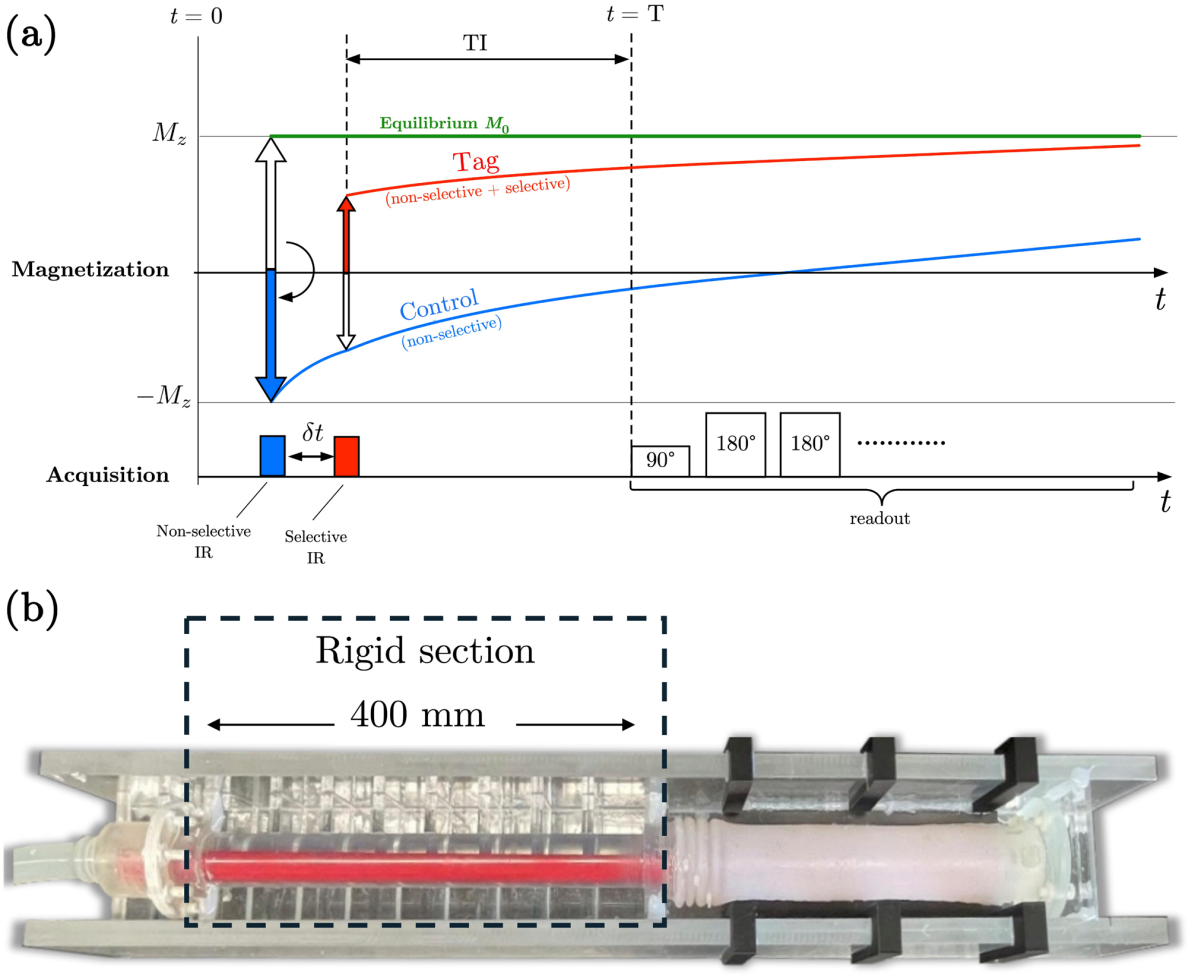
Time-SLIP sequence design and phantom flow setup. In (**a**) TimeSLIP sequence diagram illustrating magnetization evolution for the Tag condition (red curve; selective + nonselective inversion pulses) and the Control condition (blue curve; nonselective inversion pulse only). (**b**) Sealed water phantom connected to a computercontrolled pump to simulate bulk flow (provided by the Sanchez Lab, UC San Diego, Dept. of Mechanical and Aerospace Engineering). Panel (**a**) is a timing diagram and is not intended to represent the spatial configuration of the phantom shown in panel (**b**)

The Time-SLIP sequence, illustrated in Fig. 1a, selectively tags CSF to visualize its flow dynamics by combining non-selective and spatially selective inversion recovery (IR) pulses. A non-selective inversion pulse is first applied, inverting longitudinal magnetization (*M*_*z*_) across the entire imaging field-of-view, including static tissue and CSF, to negative magnetization (−*M*_*z*_). After a defined inversion time (TI), a selective inversion pulse is applied over a predefined tagging region to invert only the spins of interest, creating two signal pathways: the Control (blue curve) where only the non-selective inversion is applied and the Tag (red curve) where both selective and non-selective inversions are applied. This approach enables clear visualization of tagged fluid movement, with the tagged fluid remaining bright while the background signal is suppressed near its null point [15, 16, 27]. As the spins recover toward equilibrium (green line), the tagged fluid including CSF follows a different recovery trajectory than static tissue, and the signal increase ratio (SIR) is calculated as (Tag − Control) / *M*_0_, where *M*_0_ is the equilibrium signal. For semi-quantification direct acquisition of *M*_0_ is not strictly necessary, as it can be approximated by the control image at the longest acquired TI when longitudinal recovery is nearly complete. For CSF at 3T (T_1_ ≈ 3000 ms), this corresponds to roughly 5–7 seconds after inversion to achieve about 80–90% recovery. The schematic highlights the timing of inversion pulses, the distinct recovery curves of tagged and control signals, and the subsequent imaging readout, which is usually performed using a 2D or 3D single-shot fast spin echo (SSFSE) acquisition.

### CSF outflow models

Evolution of tagged signal in Time-SLIP has been previously modeled with continuous analytical functions as function of TI. This approach employs a Г-variate function, originally adapted from perfusion modeling, which captures the gradual rise and decay of tagged spins as they traverse the imaging region. This model expresses the signal increase ratio SIR(TI) as a function of bolus arrival delay (Δ*t*), an apparent spin-lattice relaxation time (${\rm{T}}_{\rm{1}}^{{\rm{ app}}}$) that approximates the decay kinetics of the tagged component, and a perfusion-like scaling factor *f*:

1 $${\rm{SIR}}\left( {{\rm{TI}}} \right) = f \cdot \left( {{\rm{TI}} - {\rm{\Delta }}t} \right) \cdot {\rm{exp}}\left( { - {{\left( {{\rm{TI}} - {\rm{\Delta }}t} \right)} \over {T_1^{{\rm{app}}}}}} \right)$$

while the Г-variate function has been applied for the semi-quantification of SIR in previous studies, multiple studies have hypothesized that CSF transport and waste clearance may not governed by a single mechanism and may include rapid bulk flow along perivascular or meningeal routes and slower dispersion or perfusion-like exchange within parenchymal spaces [28–30]. The sharp, symmetric bolus profiles expected for bulk displacement are not well captured by the Г-variate function, motivating the use of a Gaussian function as an alternative description for this component:

2 $${\rm{SIR}}\left( {{\rm{TI}}} \right) = A\left[ {{\rm{exp}}\left( { - {{{{(\left( {{\rm{TI}} - {\rm{\Delta }}t} \right) - 2\tau )}^2}} \over {2{\tau ^2}}}} \right) - ex{p^{ - 2}}} \right]{1_{\left[ {{\rm{\Delta }}t,{\rm{\Delta }}t + 4\tau } \right]}}\left( {{\rm{TI}}} \right)$$

In this formulation, SIR(TI) is defined by the amplitude *A* of the Gaussian component, a time parameter corresponding to bolus arrival (Δ*t*), and a width parameter (τ) that controls the temporal spread of the Gaussian profile with indicator function **1** constraining Gaussian to 0 for TI *<* Δ*t* and TI *>* Δ*t+*4τ. Building on these two representations, we propose a bi-component model that combines the Г-variate function and Gaussian terms:

3 $$\begin{gathered}{\mathrm{SIR}}\left( {{\mathrm{TI}}} \right) = f \cdot \left( {{\mathrm{TI}} - \Delta {t_\Gamma }} \right) \cdot {\mathrm{exp}}\left( { - \frac{{\left( {{\mathrm{TI}} - \Delta {t_\Gamma }} \right)}}{{T_1^{{\mathrm{app}}}}}} \right) \hfill \\+ A\left[ {{\mathrm{exp}}\left( { - \frac{{{{\left( {\left( {{\mathrm{TI}} - \Delta {t_G}} \right) - 2\tau } \right)}^2}}}{{2{\tau ^2}}}} \right) - {e^{ - 2}}} \right]{1_{\left[ {\Delta {t_G},\Delta {t_G} + 4\tau } \right]}}\left( {{\mathrm{TI}}} \right) \hfill \\\end{gathered} $$

This composite function models the total SIR(TI) as the sum of a perfusion-like component represented by Г-variate function (Eq. 1), and a bulk-flow component represented by Gaussian (Eq. 2). By simultaneously fitting both terms, the model separates slow clearance from rapid displacement, providing a physiologically motivated framework to analyze complex CSF outflow. Including ISF and other brain waste products, where both mechanisms are present. To ensure that both models remain physiologically plausible and numerically well-behaved, each parameter (Table 1) was restricted to a range motivated by expected bolus transportation mechanics and relaxation-driven signal decay. For the Γ-variate model, which has three parameters, the perfusion-like factor and bolus delay were constrained to non-negative values consistent with the observed SIR measurements, and the apparent relaxation time was limited to a broad interval that incorporates relaxation times of CSF, ISF and other brain waste products. The bi-component model contains six nominal parameters; however, the effective degrees of freedom are reduced through physiologically motivated constraints. The apparent relaxation time of the perfusion-like term is fixed rather than fitted, reflecting the expectation that the slower-decaying component represents a mixture of long-T_1_ fluids. To preserve the physical ordering of bolus propagation, the Gaussian arrival time is constrained to precede the Γ-variate arrival time. Bounds on Gaussian amplitude and spread limit the model to waveform shapes compatible with observed SIR ranges and bolus passage time. Finally, to avoid unnecessary overparameterization, the model includes a built-in decision rule that suppresses the Γ-variate term when its inclusion does not substantially improve fit quality. In such cases, the algorithm defaults to a Gaussian only term, thereby ensuring that the bi-component model does not artificially attribute perfusion-like behavior where displacement alone provides an adequate fit.

**Table 1:** Parameters and boundaries for Г-variate and bi-component fits

Parameter			Fitting bounds	
Symbol	units	Description	Г-variate	Bi-component
*f*	s^−1^	Perfusion-like parameter	[0, SIR_MAX_]	[0, SIR_MAX_]
$\Delta {{\rm{t}}_{\rm{}}}$	ms	Perfusion-like bolus arrival time	[0, 2000]	[0, 2000] >$\Delta {{\rm{t}}_{\rm{G}}}$
${\rm{T}}_{\rm{1}}^{{\rm{app}}}$	ms	Apparent T_1_ of fluid	(0, 8000]	2000 ms (**fixed**)
A	unitless	Gaussian amplitude	–	[0, SIR_MAX_]
$\Delta {{\rm{t}}_{\rm{G}}}$	ms	Pass-throughy bolus arrival time	–	[0, 1000] **<**$\Delta {{\rm{t}}_{\rm{}}}$
${\rm{\tau }}$	s^−1^	Gaussian spread	–	[0, 2000]

### Phantom study

A phantom experiment was performed to validate the proposed Gaussian model to describe bulk flow–related signal changes in the absence of perfusion-like effects. A sealed water phantom, provided by the Sanchez Lab (UC San Diego, Department of Mechanical and Aerospace Engineering), was connected to a ViVitro SuperPump (Victoria, BC, Canada), which delivers programmable flow profiles to enable controlled simulation of bulk CSF motion. The phantom (Fig. 1b) consisted of a 400 mm rigid section, used for imaging study, and an expandable silicone section at the end to accommodate pressure variation allowing for a bulk flow dynamic at small stroke volumes. Imaging was performed on a 3T clinical scanner (Vantage Galan 3T, Canon Medical, Japan) using a 2D SSFSE Time-SLIP with the following parameters TE_*eff*_/TR = 30/5000 ms, echo train spacing (ETS) = 5 ms, and a parallel imaging (SPEEDER) factor of 2, coronal orientation, slice thickness 5 mm, FOV = 20 cm (PE) × 30 cm (RO) and matrix size = 256 (PE) × 384(RO). A Time-SLIP series was acquired with inversion times (TI) spanning 3 seconds with ΔTI = 100 ms increments, generating paired Tag and Control images. The tag pulse with a 20-mm thick slab was applied perpendicularly to both the phantom and the direction of flow. The pump was operated in oscillatory mode with a 1-second period and a stroke volume corresponding to a total fluid displacement of 20 mm. Although the flow was oscillatory rather than strictly unidirectional, each phase produced a transient bolus of tagged fluid that mimics pass-through CSF outflow making this setup suitable for validating the bulk-flow component of the model. Difference images (Tag − Control) were calculated voxel-wise; prior to subtraction, a two-dimensional median filter (3 × 3 kernel) was applied to both Tag and Control volumes to reduce noise. The control image acquired at the longest TI (4119 ms) was used as the *M*_0_ reference for SIR calculation. Signal increase ratio (SIR) curves were extracted from a spatially fixed ROI (width × height: 15 mm × 10 mm) positioned centrally along the displacement path. Given the

**Fig. 2:**
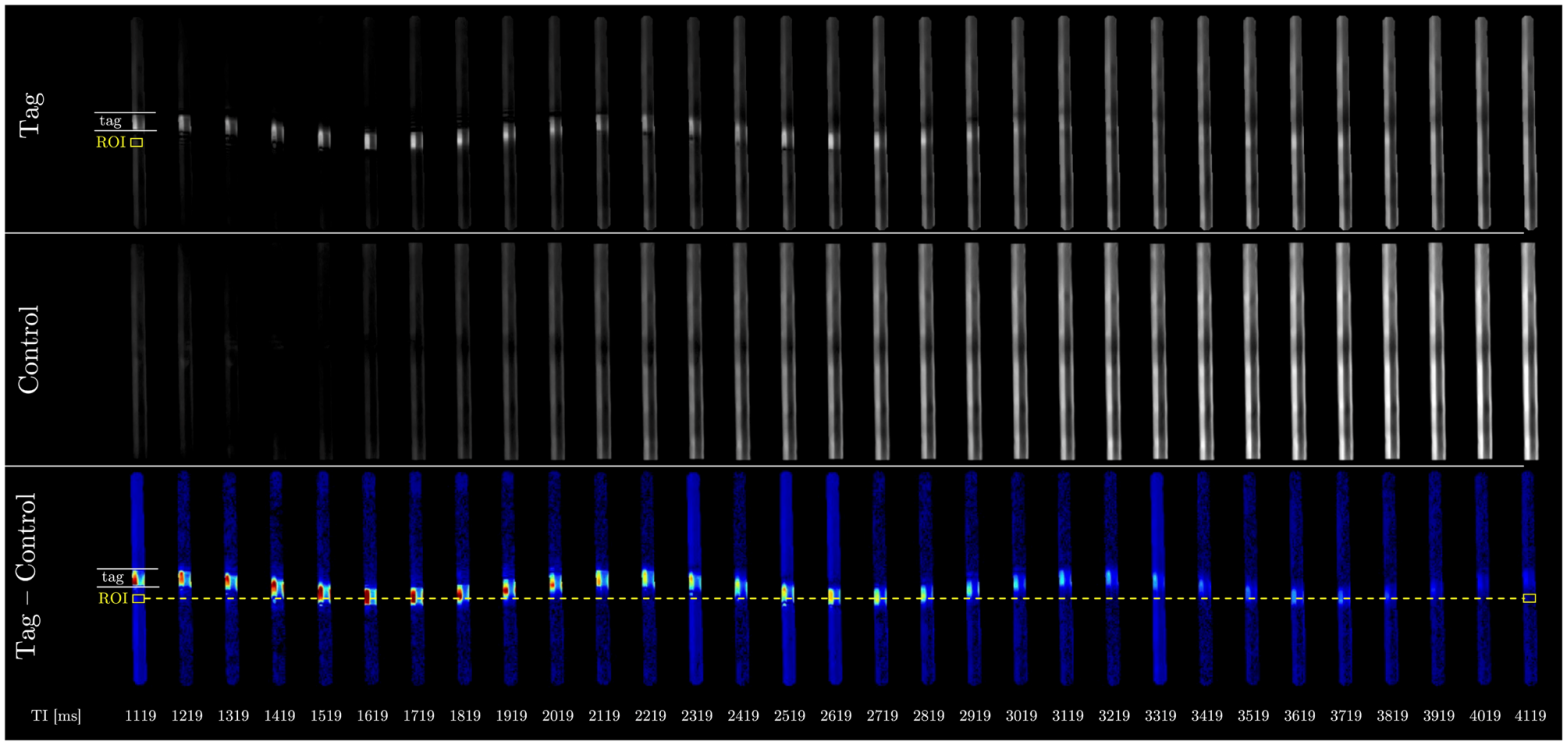
Phantom study Time-SLIP images. Top row shows Tag images; middle row shows Control images; bottom row shows subtraction maps (Tag − Control) highlighting bulk water motion. The position of the tagging pulse (white lines) and the ROI (yellow rectangle) are indicated on the first Tag image (top row) and on the subtraction maps (bottom row). The ROI position was spatially fixed, as indicated by the dotted line corresponding to the ROI center shown across all inversion times. Inversion time (TI) starts from 1119 ms and ends at 4119 ms with an increment of 100 ms in a total of 31 TIs or images

20-mm thick tag slab, the ROI sampled only a portion of the tagged bolus as it passed through the ROI. The positions of the tag pulse and ROI are indicated in Fig. 2. The effective bolus length was approximately 20 mm, corresponding to the tag slab thickness. SIR curves corresponding to each oscillatory cycle were fit using the Γ-variate model (Eq. 1) and the Gaussian model (Eq. 2), and model performance was evaluated using the coefficient of determination (R^2^), sum of squared errors (SSE), and root mean square error (RMSE).

### In-vivo study

Eight healthy volunteers: 30 ± 10 years old, range 19–48; 5 males (M), 3 females (F), were enrolled under an IRB-approved protocol and provided written informed consent. All imaging was performed on a 3 Tesla clinical scanner (Vantage Galan 3 T, Canon Medical, Japan). Time-SLIP data were acquired in the coronal orientation using a 3D SSFSE readout with zigzag centric *k*_*y*_ *k*_*z*_ Cartesian and exponential refocusing flip angles (eFA) for fast longitudinal recovery, [31] with phase encoding (PE) direction left to right and readout (RO) direction superior to inferior. Imaging parameters: TE_*eff*_/TR = 30/5400 ms, ETS = 5 ms, flip/refocusing angles = 90° / 150°, Spectral Adiabatic Inversion Recovery (SPAIR) fat suppression, FOV = 25 cm × 25 cm, acquisition matrix = 368 × 368 (interpolated to 736 × 736), SPEEDER factor = 3, and 20 contiguous slices of 1 mm thickness, ungated. For each inversion time (TI = 500, 750, 1000, 1150, 1250, 1350, 1500, 2000, and 3000 ms), a separate 3D Time-SLIP series consisting of paired Tag and Control volumes was acquired, each requiring 1 minute and 30 seconds for completion (Supporting Figure S1). The tagging slab was prescribed in an oblique-parasagittal orientation angled towards the axial plane and positioned parallel to the left side of a superior sagittal sinus. Prior to the analysis all Time-SLIP volumes were aligned using three-dimensional rigid-body (six degrees of freedom) registration in MATLAB R2025b (MathWorks, Natick, MA, USA) via *imregister* function with a multimodal mutual-information metric. The Control volume at longest TI served as the fixed reference, and transformations were applied using *imwarp* function. The fixed Control image at TI = 3000 ms was also used as the equilibrium M₀ for SIR normalization, and voxel-wise SIR was computed as (Tag – Control)/M₀. A region of interest encompassing the entire area of superior sagittal sinus (eSSS) was manually delineated on one of the Tag images to avoid tagging area. To assess whether different anatomical compartments along the parasagittal dura region exhibit systematically distinct bi-component behavior, additional sub-regions were outlined within the broader eSSS mask. These included parasagittal dura–adjacent areas (Left Middle, Left Upper, Low), their corresponding meninges lymphatic-vessel segments (LU mLV, Low mLV), the Right Upper and Right Middle regions (RU, RM), and the central Superior Sagittal Sinus (SSS). Subtraction colormaps (Tag – Control and SIR) were generated for visual inspection. The mean SIR for each subject was fitted separately to the Г-variate function (Eq. 1), Gaussian (Eq. 2), and the bi-component model (Eq. 3). Similar to the phantom study the goodness-of-fit was evaluated using R^2^, SSE, and RMSE metrics. All analyses were performed using custom scripts developed in-house in MATLAB R2025b (MathWorks, Natick, MA, USA). Bi-component model parameters were estimated using bounded nonlinear least-squares optimization (Curve Fitting Toolbox, fit, trust-region-reflective algorithm).

## Results

### Phantom study

**Fig. 3:**
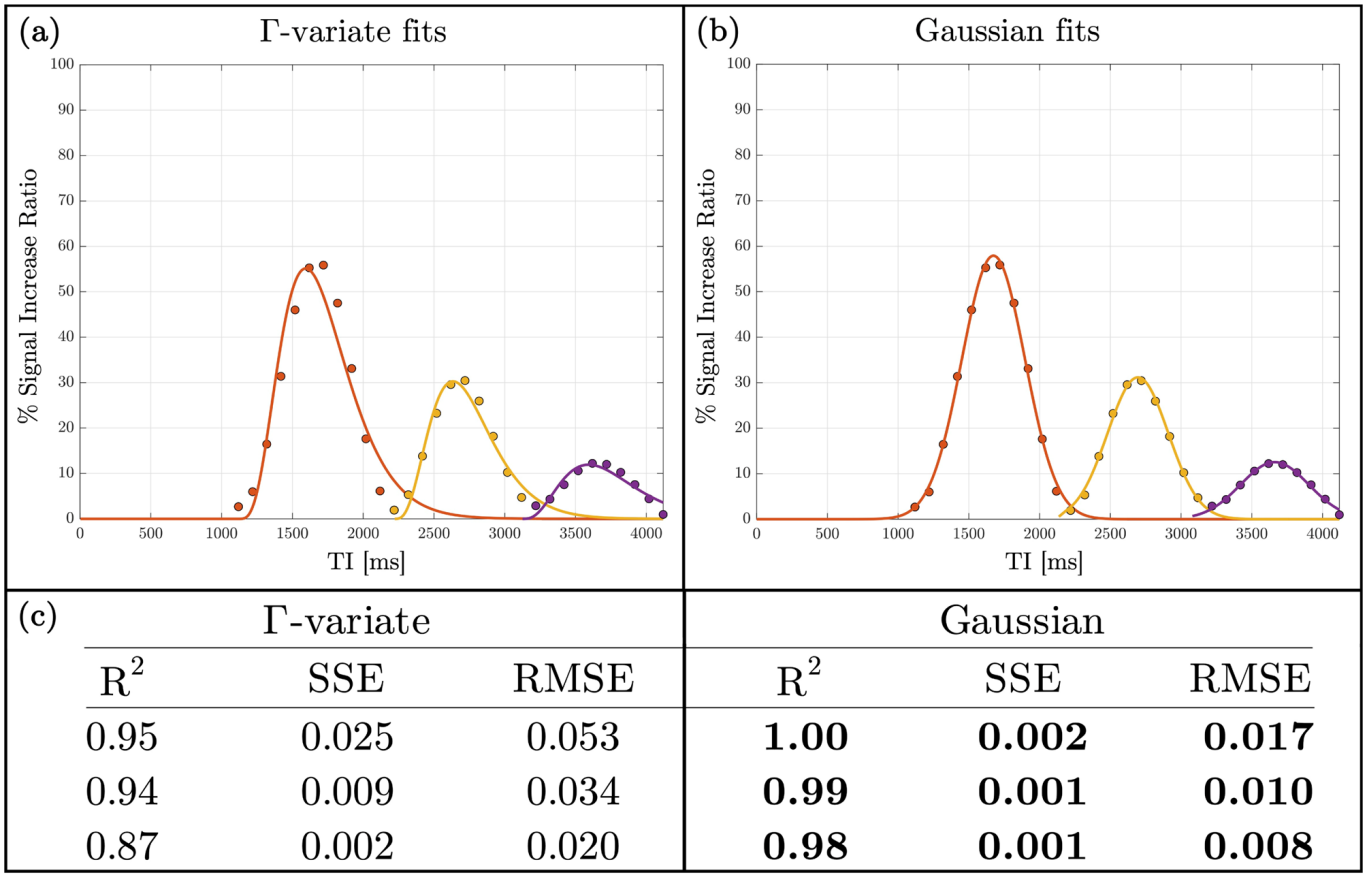
Comparison of Gaussian and Γ-variate fits in phantom data. (**a**) Γ-variate and (**b**) Gaussian model fits for each bulkmotion cycle in the phantom study. (**c**) Corresponding goodness-of-fit metrics (R^2^ – R-squared or coefficient of determination, SSE – sum of squared errors, RMSE – root mean square error); bold values indicate the better fit

The phantom Time-SLIP Data are shown in Fig. 2, where the top two rows present the Tag and Control images for three successive oscillation cycles and the bottom row shows the corresponding subtraction colormaps, providing a clear visualization of bulk flow dynamics. In Fig. 3, panel (a) shows the Γ-variate model fits and panel (b) the Gaussian fits to the voxel-averaged signal increase ratio (SIR) curves for each cycle. Quantitative metrics table in Fig. 3c (R^2^, SSE, RMSE) demonstrates that the Gaussian model outperforms the Γ-variate fit in all three cycles, with higher R^2^ and lower SSE and RMSE values (highlighted in bold). For Г-variate fits in the phantom, the apparent *T*_1_ constant converged to ${\rm{T}}_{\rm{1}}^{{\rm{app}}}{\rm{ = 128 \pm 18 ms}}$ for distilled water at 3T, which is an order of magnitude shorter than the known ${{\rm{T}}_{\rm{1}}}$ of distilled water ≈3000 ms [32]. When the Г-variate was constrained to a *T*_1_  = 3000 ms, the fit quality deteriorated markedly (Supporting Fig. 2). By contrast, the Gaussian function provided an adequate description of the compact, symmetric peak produced by bulk water displacement.

### In-vivo human meninges study

**Fig. 4:**
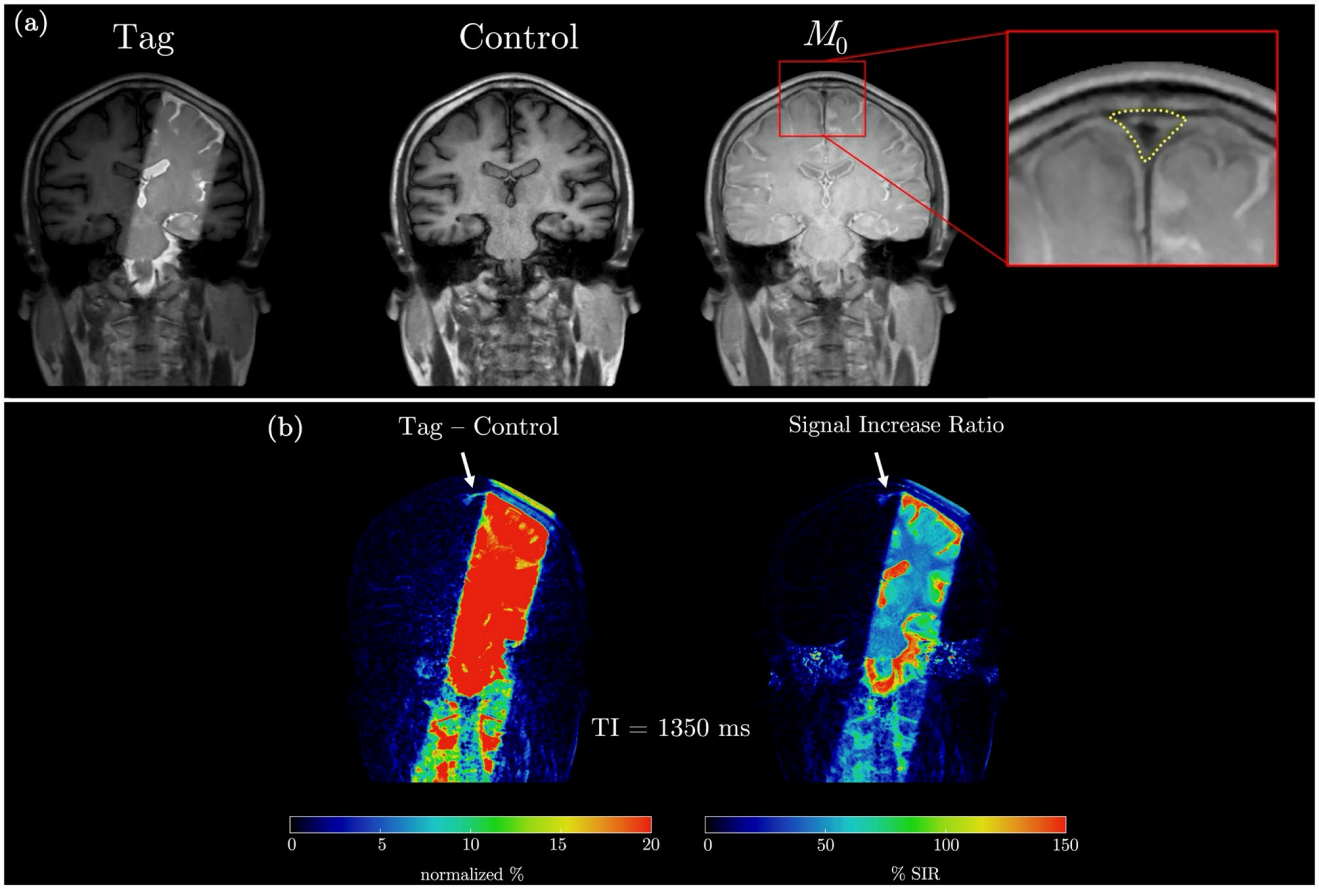
In-vivo Time-SLIP images of the superior sagittal sinus region. Tag, Control, and equilibrium (*M*_0_) images are shown in panel (**a**), with the region of interest (yellow outline) selected for quantifying CSF outflow. Panel (**b**) presents the subtraction map (Tag – Control) and the corresponding signal increase ratio (SIR) map at inversion time TI = 1350 ms, where CSF outflow is highlighted by the arrow

**Fig. 5:**
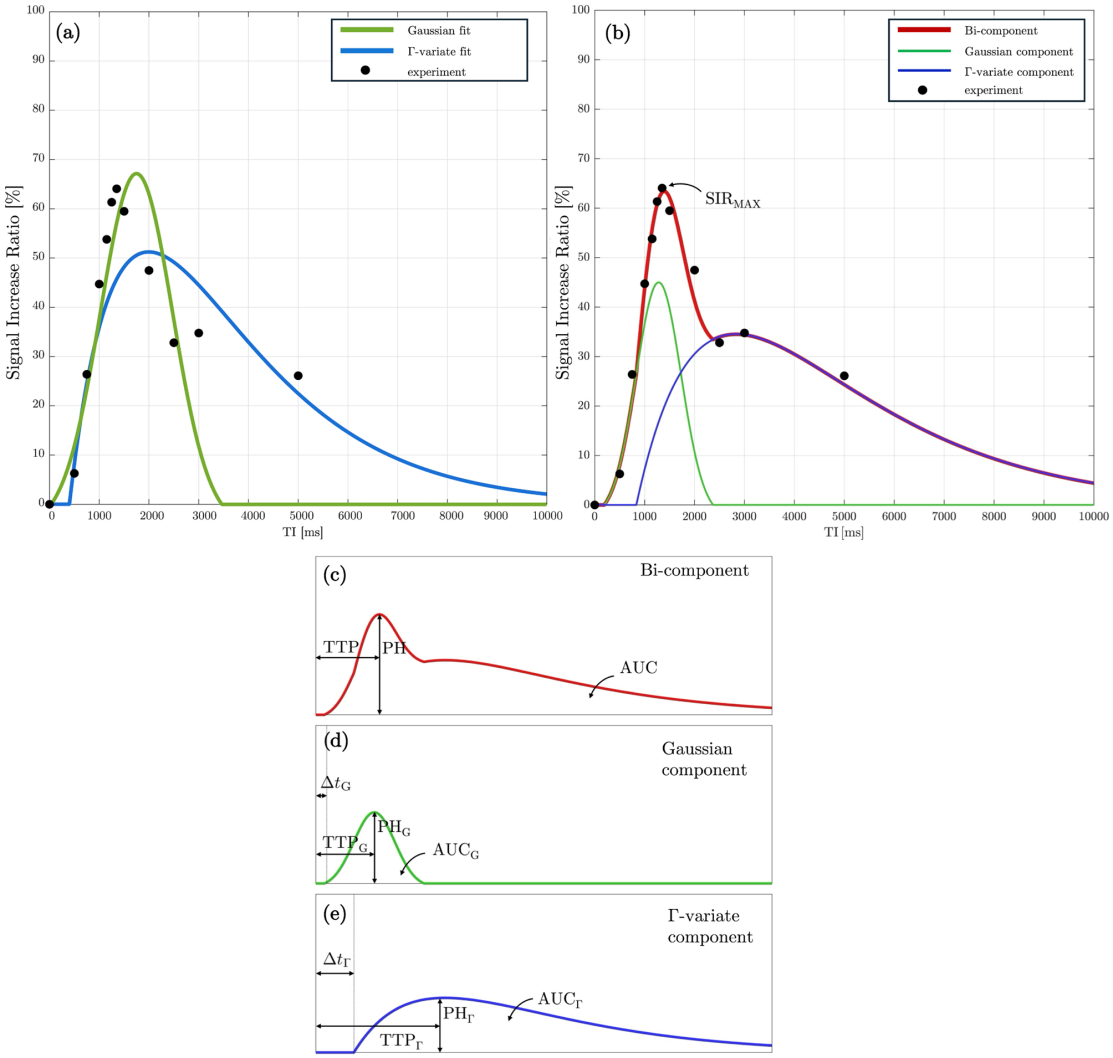
Bi-component modeling of CSF outflow signal. (**a**) Single-component fits using Gaussian (green) and Γ-variate (blue) functions for a representative participant. (**b**) Bi-component fit (red) combining Gaussian (green) and Γ-variate (blue) components, with the experimental data shown as black dots. (c–e) individual components of the bi-component model, with annotated quantitative parameters including max measured raw signal increase ratio (SIR_MAX_), time-to-peak (TTP), peak height (PH), area under the curve (AUC), and arrival delays (Δ*t*)

**Fig. 6:**
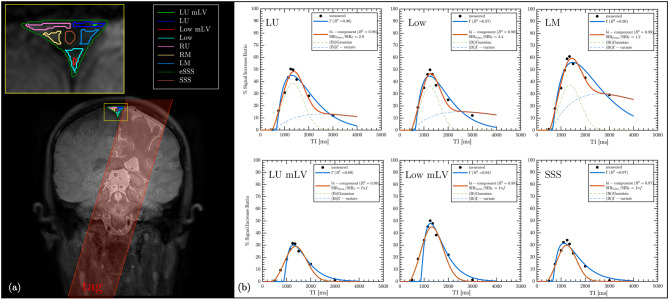
Placement of ROIs and representative SNR curves. (**a**) Manually defined regions of interest (ROIs) overlayed on top of the tag image, including left Upper (LU), left Middle (LM), Low, right Upper (RU), right Middle (RM), LU and Low meninges lymphatic vessels (mLvs), superior sagittal sinus (SSS), entire SSS (eSSS). (**b**) signal increase ratio (SIR) fits with Г-variate (blue), bi-component (red) model fits for six selected ROIs

**Fig. 7:**
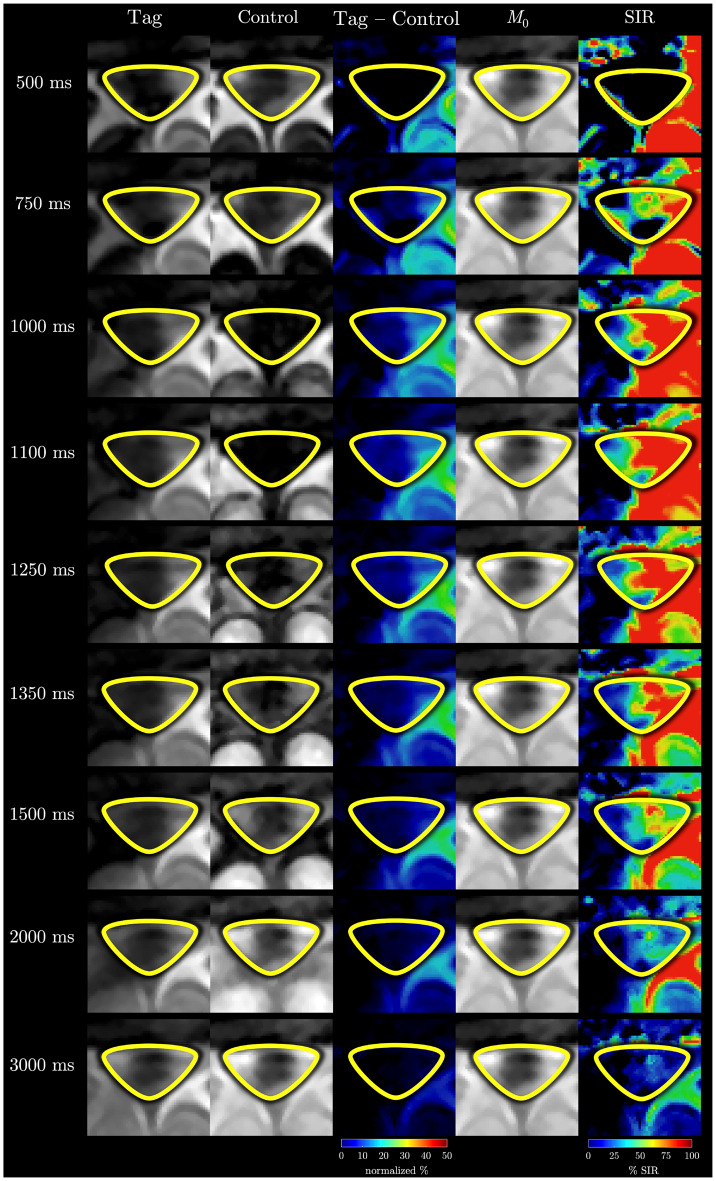
Temporal evolution of CSF outflow signal in the superior sagittal sinus. Mosaic zoomed-in view of Tag, Control, subtraction (Tag – Control) colormaps, equilibrium (*M*_0_) images, and signal increase ratio (SIR) colormaps at multiple inversion times (TIs) ranging from 500 to 3000 ms, illustrating the temporal evolution of the CSF outflow signal within the superior sagittal sinus region of interest

**Fig. 8:**
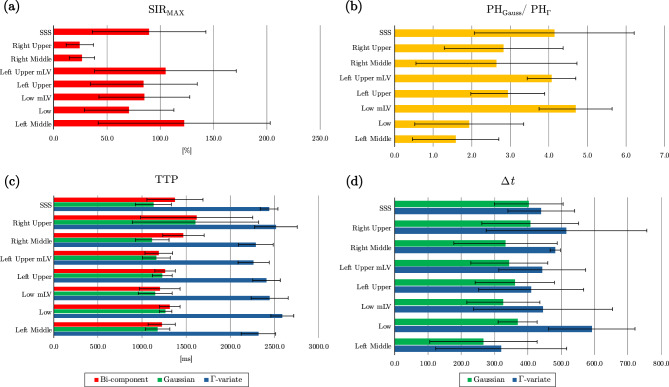
Bar charts of selected bi-component model metrics across ROIs. (**a**) Max measured raw signal increase ratio (SIR_MAX_) without fit. (**b**) ratio of peak heights of the Gaussian and Г-variate components (PH_Gauss_ / PH_Г_). (**c**) Time-to-peak (TTP) for bi-component model (red), Gaussian component (green), Г-variate component (blue). (**d**) Arrival delay ($\Delta {\rm{t}}$) for Gaussian (green) and Г-variate (blue) components. All charts have the error bars representing standard deviation

Tag, Control, and equilibrium (*M*_0_) image of a single slice for one of the subjects with zoomed-in region including the manually defined SSS ROI used for quantitative analysis are shown in Fig. 4a. Normalized subtraction colormaps (Tag – Control) and the corresponding Signal SIR colormap at TI = 1350 ms for the same subject (Fig. 4b) highlight the appearance of tagged fluid within the ROI. The outflow is visually observable in the colormaps, as indicated by the white arrow. Model fits of the measured SIR for one of the participant are presented in Fig. 5. Panel 5a shows Gaussian (green) and Г-variate (blue) fits – single-component approach. Bi-component fit (thick red) is shown in panel 5b that also shows Γ-variate (blue) and Gaussian (green) components. In panels 5c-e each of the component of bi-component fit are shown individually with the annotated quantitative metrics. The bi-component model provided a superior fit, achieving a higher R^2^ value compared to the Γ-variate function. Across subjects, the bi-component fit parameters showed systematic regional variation. Representative example for one of the subject is shown in Fig. 6. In 6a the placement of each ROIs is demonstrated and the fits corresponding to 6 different regions closest to the tagging pulse are shown in 6b. ROIs adjacent to the parasagittal dura (e.g LU Low and LM) exhibit a distinct slowly decaying second component with substantial signal at long TIs that is well fit by the Γ-variate function. On the other hand, meninges lymphatic-vessels ROIs (LU mLV, LOW mLV) exhibit a symmetric Gaussian peak with almost no late-time tail resulting in fits without the perfusion-like component. The temporal evolution of the tagged CSF signal is further illustrated in the zoomed mosaic view in Fig. 7, which presents Tag, Control, subtraction colormaps (Tag – Control), *M*_0_, and SIR colormaps across inversion times ranging from 500 to 3000 ms. The SSS ROI boundary is overlaid in yellow on the top of each image. In both subtraction and SIR colormaps minimal signal is observed at the smallest TI = 500 ms with a pronounced peak at mid-range TIs (1150–1250 ms), and a gradual decay at longer TIs. Average quantitative parameters describing the temporal and amplitude characteristics of the SIR, including time-to-peak (TTP), full-width at half-maximum (FWHM), pelak height (PH), and area under the curve (AUC), together with model fit metrics (Rar, RMSE, SSE), for the Γ-variate, Gaussian, and bi-component model fits are provided in Table 2. While Gaussian fit alone was less accurate than the Γ-variate model, combining both components in the bi-component model provided the best fit, yielding higher R^2^ values and lower SSE and RMSE. Detailed results per subject for eSSS ROI are provided in Supporting Table S1, which includes demographic data, maximum raw SIR values, and model specific parameters. For the single-component fits, this includes bolus delay (Δ*t*) for both the Gaussian and Γ-variate models and ${\rm{T}}_{\rm{1}}^{{\rm{ app}}}$ for the Γ-variate fit. For the bi-component model, parameters for each component are reported separately, including TTP, Δ*t*, and the PH ratio between the Gaussian and Γ-variate components. For the rest of the ROIs average values for all the parameters of the bi-component model are provided in Supporting Table S2 with some of the metrics further visualized using bar charts in Fig. 8. Higher PH ratios between the Gaussian and Γ-variate components as expected were observed for both mLV regions as well as SSS. To further assess whether bi-component parameters differed systematically across anatomical regions, we performed a repeated-measures ANOVA across all ROIs. Given the limited sample size, these statistical results are intended as semi-exploratory guidance for the interested reader rather than strong claims. The corresponding post-hoc comparisons are visualized in Supporting Figure S3, where ROI-to-ROI differences in each metric are displayed using directional arrows and color-coded significance after Benjamini–Hochberg false-discovery-rate (BH–FDR) correction. Solid lines mark statistically significant effects, whereas dashed lines denote near-significant trends (0.05 < *p* < 0.10)

**Table 2:** Average quantitative parameters describing the temporal and amplitude characteristics of the signal increase ratio, along with goodness-of-fit metrics, for the Γ-variate, Gaussian, and bi-component model fits

	Г-variate	Gaussian	Bi-component
TTP [ms]	1237 ± 101	1368 ± 95	1262 ± 87
FWHM [ms]	1513 ± 298	1174 ± 84	1243 ± 229
PH [%]	64 ± 18	67 ± 17	66 ± 17
AUC [%⋅s]	100 ± 17	81 ± 16	118 ± 24
R^2^	0.93 ± 0.05	0.86 ± 0.06	0.98 ± 0.01
SSE	0.057 ± 0.084	0.080 ± 0.038	0.014 ± 0.015
RMSE	0.076 ± 0.050	0.104 ± 0.026	0.055 ± 0.025

TTP: time-to-peak, FWHM: full width at half maximum, PH: peak height, AUC: area under curve, R^2^: R-squared or coefficient of determination, SSE: sum of squared errors, RMSE: root mean square error

## Discussion

The phantom experiment provides a reference condition in which only bulk displacement occurs. Under this controlled setting, the Gaussian model captures the compact and symmetric signal peak more accurately than the gamma-variate, which requires an implausibly short apparent ${\rm{T}}_{\rm{1}}^{{\rm{ app}}}$ to approximate the same data. This establishes the Gaussian function as the more appropriate descriptor of bulk fluid motion and serves as a controlled validation prior to its application in-vivo. However, while the phantom experiments primarily aid in interpreting model behavior under simplified and idealized conditions, in-vivo CSF transport is inherently more complex. Thus, the use of phantom results to support physiological interpretation of the Gaussian component should be made with caution, and extrapolation of these findings to in vivo conditions is subject to limitations.

Application of the bi-component model to Time-SLIP data from healthy volunteers revealed clear improvements in fitting accuracy compared to single-component model. The motivation for this model originates from multiple studies suggesting that CSF clearance involves more than one transport mode, with rapid convective outflow along meningeal or perivascular routes and slower exchange or dispersion in mixture of ISF-CSF compartments. Our measured SIR curves similarly deviate from the unimodal profiles expected for a single clearance process, instead showing an early sharp rise followed by a prolonged tail that aligns with overlapping fast and slow phases.

Our hypothesis is that the fast Gaussian component represents bulk CSF outflow, while the slower Γ-variate term likely reflects perfusion like transport involving ISF, CSF, and metabolic wastes. Recent high-resolution MRI findings by Yoo et al. demonstrated functional mLVs along the superior sagittal and transverse sinuses, suggesting that the slower component observed in our study may correspond to lymphatic-associated drainage within or adjacent to these mLV pathways [33]. Physiologically, CSF, having lower viscosity and density relative to ISF solutes or cellular debris would favor rapid clearance, while the mixture of waste products may exhibit a delayed washout, consistent with the broader temporal profile described by the Γ-variate component.

The Γ-variate model alone produced reasonable fits, but its apparent ${\rm{T}}_{\rm{1}}^{{\rm{ app}}}$ estimates (Supporting Table S1) deviated substantially from known CSF relaxation values, reducing its physiological relevance. While a bi-component formulation has greater flexibility than a single Γ-variate function, several aspects of the implementation prevent it from simply overfitting the data. The two components are not treated symmetrically: the Gaussian term is constrained by physiologically plausible bounds on its arrival time and width, while the slower component uses a fixed ${\rm{T}}_{\rm{1}}^{{\rm{app}}}$ rather than an additional free relaxation parameter. Moreover, the model includes an explicit model-selection step in which a Gaussian-only fit is computed in parallel; the Γ-variate contribution is retained only if it provides a clear improvement in goodness-of-fit. When it does not, the algorithm automatically suppresses the perfusion-like component and defaults to a single-component model. This behavior was confirmed in the phantom experiment, where only bulk displacement is present, and the algorithm consistently converged to a purely Gaussian fit. Together, these features allow the bi-component model to reflect true physiological heterogeneity while limiting the risk of overfitting.

Across subjects, the regional behavior of the bi-component model was consistent with physiological expectations. ROIs corresponding to lymphatic vessels and the superior sagittal sinus were dominated by rapid bulk outflow and either showed complete suppression of the perfusion-like Γ-variate term or, when present, exhibited a substantially higher Gaussian-to-Γ PH ratio. In contrast, ROIs placed on LU, LM, and Low- parasagittal dura demonstrated a clear and physiologically plausible slow-decaying tail captured by the Γ-variate component. As expected from the geometry of the experiment, ROIs closer to the tag (e.g., left-sided ROIs) demonstrated shorter TTP than right-sided ROIs. Although these regional trends are promising, perfect binary separation between “bulk-flow–dominated” and “exchange-dominated” ROIs is not expected. Right-sided ROIs (RU, RM) showed consistently lower SIR amplitudes, due to greater distance from the left tag; in these regions the slow component often falls near or below the noise level, causing the algorithm to default to a Gaussian-only fit. Conversely, the occasional presence of a small Γ-variate tail in mLV ROIs is physiologically reasonable, as these vessels interface with parasagittal dura spaces and are unlikely to behave as perfectly isolated single-compartment.

Several recent studies have emphasized the importance of distinguishing CSF outflow from venous blood flow in the SSS [34, 35]. In this context, we carefully examined whether venous signal contamination could influence the Time-SLIP measured SIR. In our protocol, such contamination is unlikely for two reasons. First, the SSFSE readout with an ETS of 5 ms inherently limits the refocusing of rapidly moving venous spins, especially in the superior sagittal sinus, resulting in intravoxel dephasing and signal attenuation [36]. Second, we conducted a validation experiment incorporating a *T*_2_ Preparation (*T*_2_ Prep) module between the Time-SLIP tagging and acquisition (Supporting Figure S4a). The MLEV (Malcom-Levitt)-4–based *T*_2_ Prep was designed to selectively attenuate short-T₂ venous-like components while preserving long-*T*_2_ CSF-like signals [37, 38]. A three-component phantom consisting of tap water, milk, and cream (Supporting Figures S4b), representing a broad range of *T*_2_ values from long-*T*_2_ CSF-like water (≈1.5–2.0 s) to short-*T*_2_ venous-like 2% fat milk (≈25–75 ms) [39–42]. Results from both simulations and experimental measurements (Supporting Figure S5) showed consistent behavior: the *T*_2_ Prep with TE = 70 ms attenuated short-*T*_2_ species by approximately 75–80%, while long-*T*_2_ water retained more than 90% of its baseline signal. Finally, in-vivo validation in a healthy volunteer (Supporting Figure S6) demonstrated nearly identical Time-SLIP SIR curves for data acquired with and without the *T*_2_ Prep, differing by only ≈8% in maximum SIR. These combined findings confirm that the Time-SLIP signal measured in the SSS predominantly reflects CSF outflow and is not contaminated by venous blood.

Several limitations should be acknowledged. Although our current phantom validates the bulk-flow component of the model, a dedicated dual-compartment phantom would allow controlled testing of both components simultaneously. Developing such a system represents an important future direction to further support model robustness. In addition, the temperature of the phantom contents was not measured directly and was assumed to be close to the MRI scanner room temperature; temperature-dependent variations in water T1 have been reported and may influence quantitative estimates [43]. In-vivo Time-SLIP acquisitions were ungated, meaning cardiac and respiratory pulsation may introduce variability in the observed signal. In ungated acquisitions, the timing of the tagging pulse may not be phase-aligned with underlying physiological motion, which could influence the estimated contribution of the Gaussian component and limit measurement stability or reproducibility across scans. Incorporating cardiac and/or respiratory gating in future studies may help address this limitation, improve the consistency of parameter estimates, and facilitate clearer association of the observed signal dynamics with physiological cycles. Test–retest repeatability was not evaluated in this study, as all inversion times were acquired within a single session. The applicability of the bi-component model to pathological conditions has not yet been evaluated. All the study participants are young (below 50 years old). CSF outflow metrics may differ if sleep and lifestyle factors are controlled, as this study focused primarily on the technical feasibility of the method rather than these influences. Finally, the validation in a larger cohort will be important to establish robustness and determine whether this framework can detect age or disease related changes in CSF outflow.

## Conlcusion

In the present study, we demonstrate that a bi-component model framework applied to the analysis of Time-SLIP data enables improved characterization of CSF outflow compared to previously utilized the single-component approach. By combining a Gaussian term for fast bulk flow with a Γ-variate term for slower perfusion-like transport, the model captures distinct clearance phases and provides physiologically interpretable parameters. Validation in a controlled phantom confirmed the Gaussian term’s suitability for bulk motion, and application in healthy volunteers showed superior fitting accuracy of the combined model. These findings establish a methodological basis for future investigations of age- and disease-related changes in CSF outflow metrics and pathways.

## Electronic supplementary material

Below is the link to the electronic supplementary material.

Supplementary Material 1

## Data Availability

The datasets generated and/or analyzed during the current study are available from the corresponding author on reasonable request.

## Abbreviations

Time-SLIP: Time-Spatial Labeling Inversion Pulse
*T*_2_ Prep: T_2_ Preparation
CSF: Cerebrospinal Fluid
ISF: Interstitial Fluid
SIR: Signal Increase Ratio
SSE: Sum of Squared estimate of Errors
RMSE: Root Mean Square Error
