# Supplementary material for: Bi-component modeling of cerebrospinal fluid outflow using Time-SLIP MRI
Source: Fluids Barriers CNS. 2026 Mar 12;23:61. doi: 10.1186/s12987-026-00791-9 (PMC13097922; doi:10.1186/s12987-026-00791-9)
Supplement: Supplementary file 1 — Supplementary Material 1 [file 12987_2026_791_MOESM1_ESM.pdf]

SUPPLEMENTAL MATERIAL FOR REVIEW:

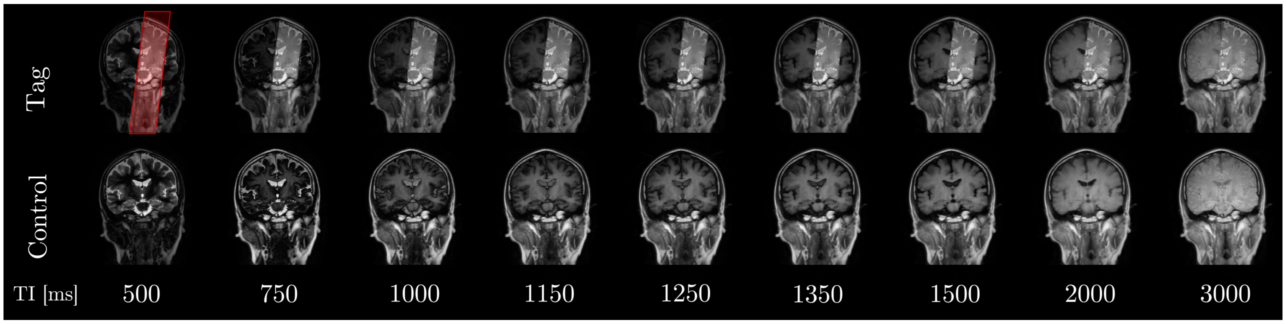

Supporting Figure S1: Mosaic view of Tag and Control Time-SLIP images of the brain acquired at multiple inversion times (TIs) ranging from 500 to 3000 ms. The orientation of tagging pulse is highlighted in red on the first Tag image (inversion time TI = 500ms.)

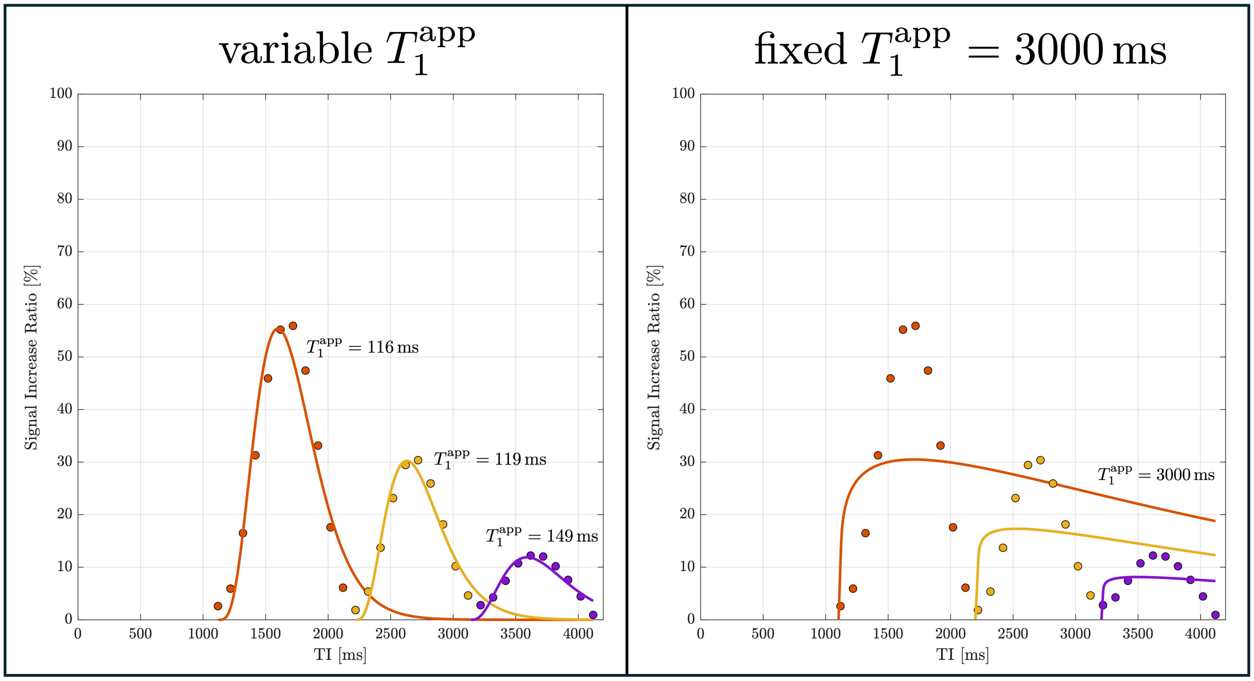

Supporting Figure S2. Γ-variate fits in the phantom experiment. Left: fit with the variable T_1^{app} yields values (116–149 ms) for water at 3T. Right: constraining $T_1^{\mathrm{app}} = 3000$ ms results in markedly degraded fits.

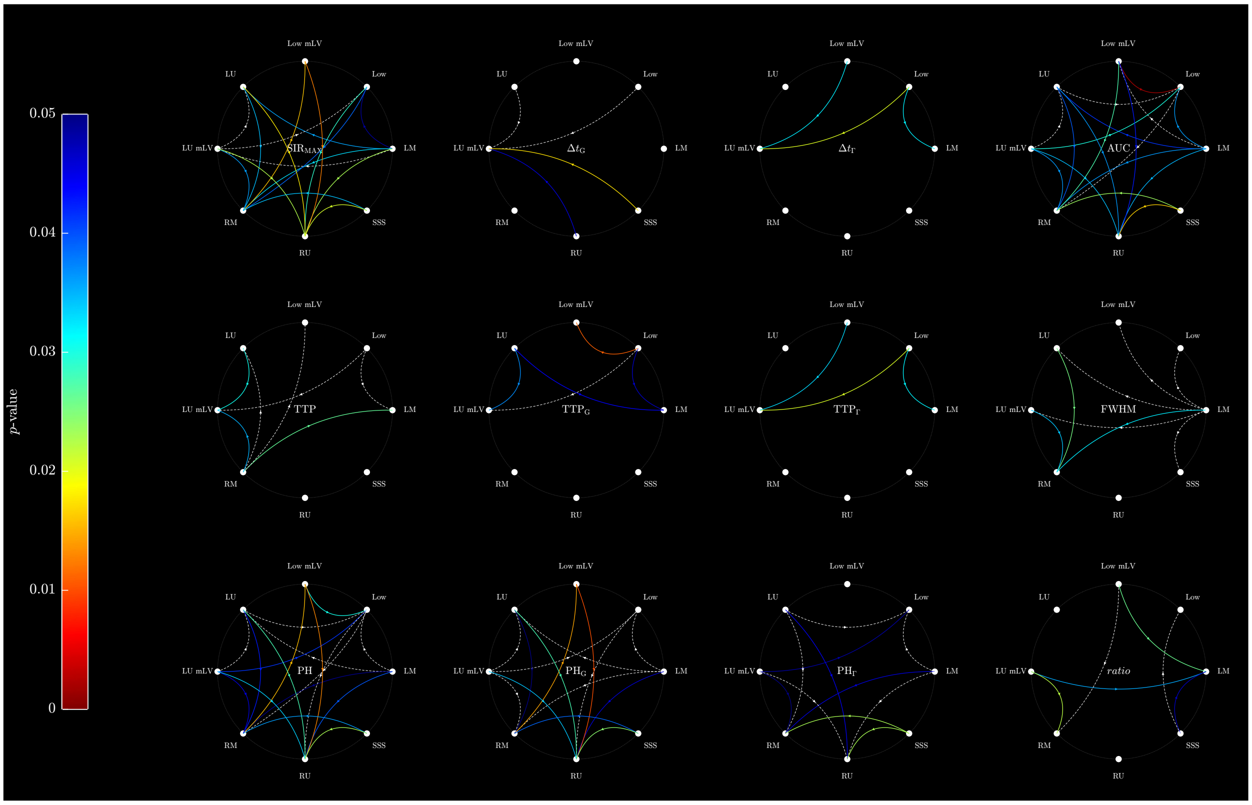

Supporting Figure S3. Post hoc pairwise comparisons of bi-component model metrics: max measured raw signal increase ratio (SIR_{MAX}), arrival delay for Gaussian (Δt_G) and Γ-variate (Δt_Γ) components, area under the curve (AUC), bi-component fit time-to-peak (TTP), Gaussian (TTP_G), Γ-variate (TTP_Γ), full-width at half-maximum (FWHM), bi-component fit peak height (PH), Gaussian (PH_G), Γ-variate (PH_Γ), and the ratio PH_G/PH_Γ – across ROIs, following repeated-measures ANOVA. Each panel depicts all post-hoc ROI-to-ROI comparisons for a given metric. Solid lines indicate statistically significant differences after Benjamini-Hochberg false-discovery-rate (BH-FDR) correction, with line color representing the corrected p-value (color bar at left, warmer colors indicate stronger differences). Arrow direction denotes the orientation of the effect, pointing from the ROI with the larger mean value toward the ROI with the smaller mean. Dashed lines represent trends ($0.05 < p < 0.10$), included for exploratory interpretation given the modest sample size (N=8).

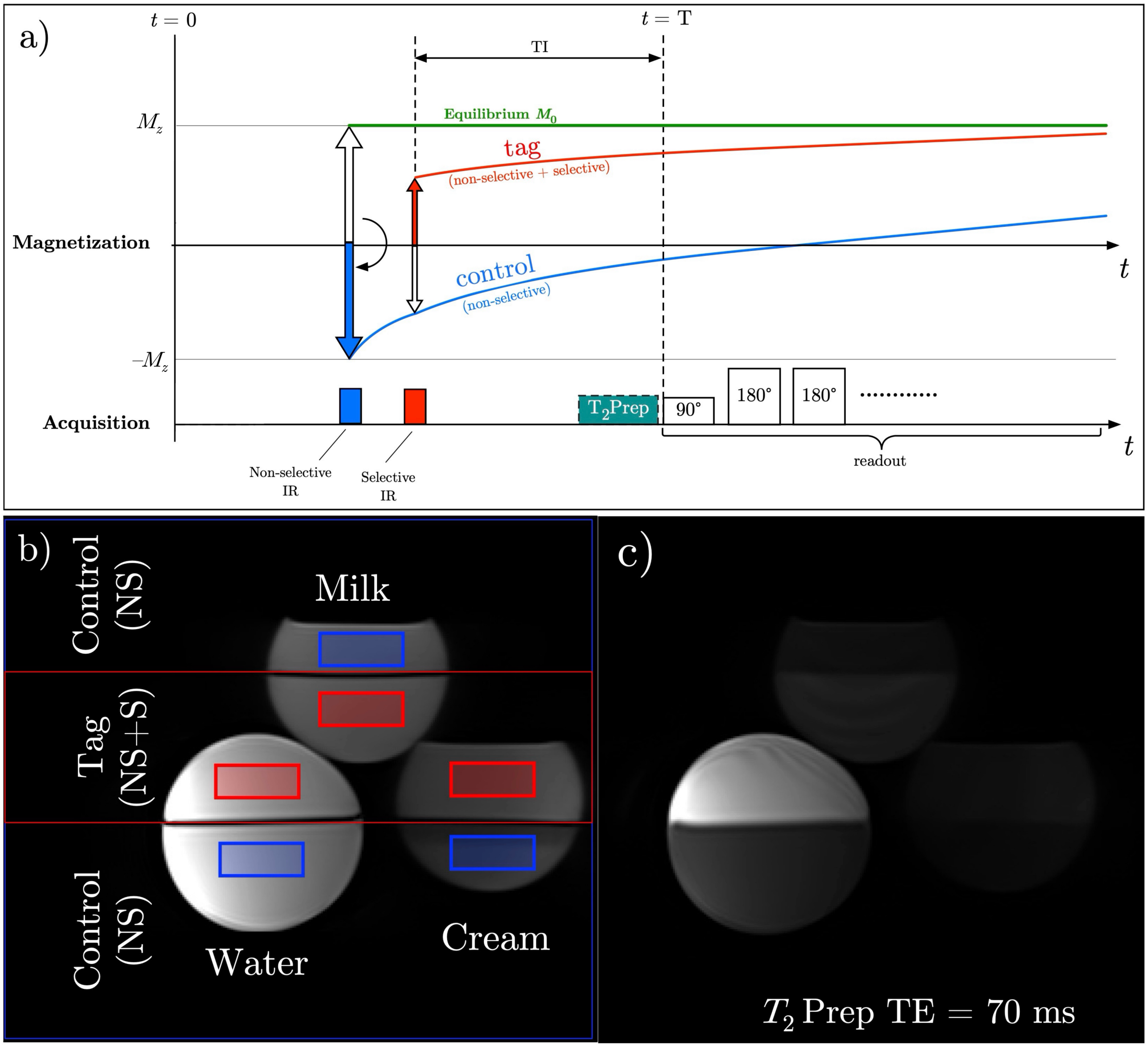

Supporting Figure S4. In (**a**) is the Time-SLIP acquisition diagram with an MLEV-4 T_2 Prep module (teal rectangle) followed by single-shot fast spin echo (SSFSE) readout. Baseline image of the three-component phantom in (**b**) acquired without T_2 Prep at inversion time (TI) = 2000 ms. In (**c**) same phantom image acquired with T_2 Prep (TE = 70 ms) also at TI = 2000 ms, demonstrating strong suppression of short-T_2 components (milk, cream) while preserving the long-T_2 water signal.

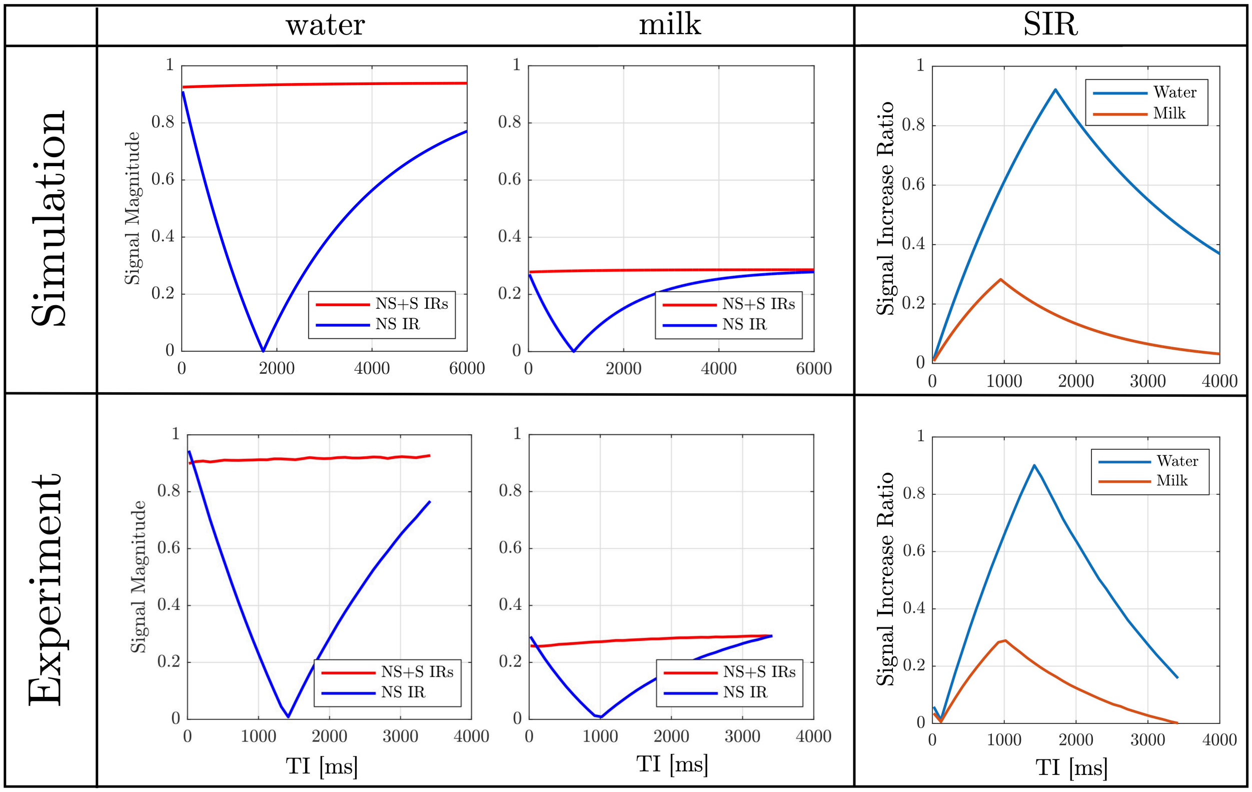

Supporting Figure S5. Simulated (top) and experimental (bottom) Time-SLIP signals for water and milk normalized to M_0 in first two columns and Signal Increase Ratio in the third column. The T_2 Prep (TE = 70 ms) attenuates short-T_2 species by approximately 75 – 80%, with less than 10% loss for long-T_2 components, confirming selective suppression of venous-like signal while maintaining CSF-like signal integrity.

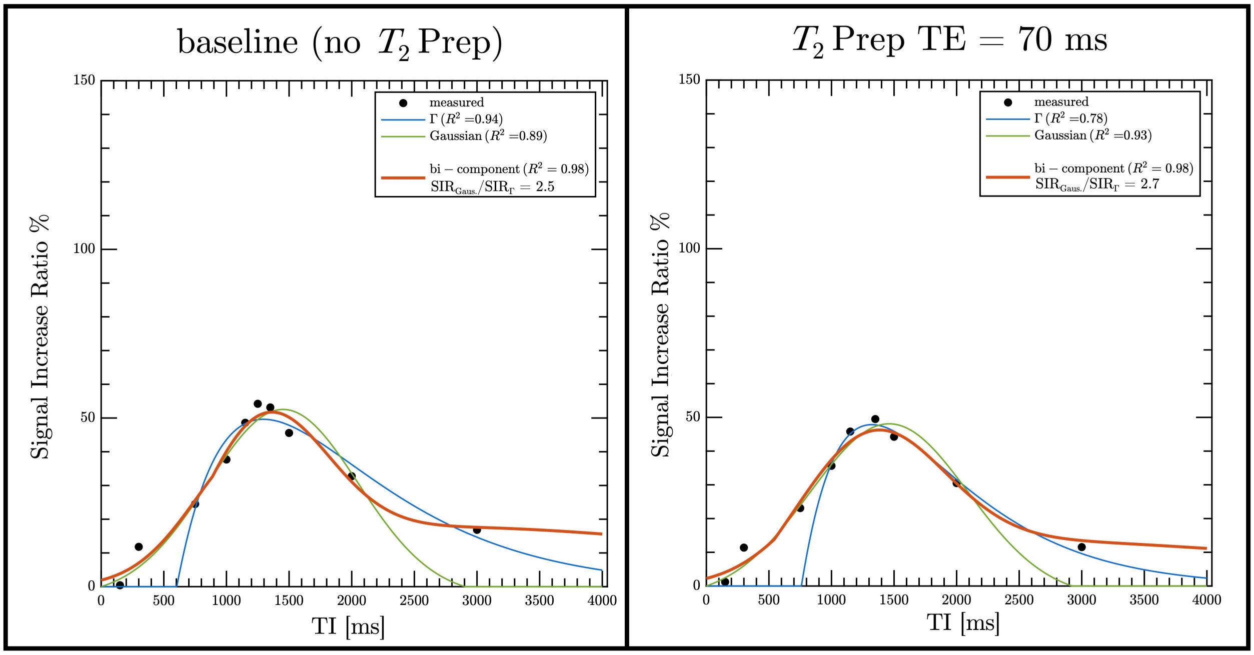

Supporting Figure S6. *In-vivo* Time-SLIP signal increase ratio (SIR) curves measured for Superior Sagittal Sinus ROI with fits. Plot (a) for data acquired without and plot (b) with T_2 Prep module (TE = 70 ms). Nearly identical profiles, with only an $\approx 8\%$ reduction in SIR, indicate that venous blood contribution to the measured signal is minimal.

Supporting Table S1. Detailed per-subject data including demographic information (age, gender) and the maximum measured raw Signal Increase Ratio (SIR). Subsequent rows present quantitative metrics (TTP – time-to-peak, FWHM – full-width at half-maximum, PH – peak height, AUC – area under the curve) and model specific parameters with the corresponding goodness-of-fit metrics (R^2 – R-squared or coefficient of determination, RMSE – root mean square error, SSE – sum of squared errors) for the Gaussian, Γ-variate, and bi-component model fits.

subject	age [years]	gender	SIR_{MAX} [%]
1	36	F	74
2	28	M	105
3	19	M	57
4	27	F	77
5	19	M	53
6	28	F	63
7	34	M	73
8	48	M	49
mean ± SD	30 ± 10	5M & 3F	69 ± 18

	Γ-variate fit						Goodness of fit		
subject	TTP [ms]	FWHM [ms]	PH [%]	AUC [%·s]	Δt [ms]	T_1 [ms]	R^2	SSE	RMSE
1	1073	1197	71	94	584	491	0.90	0.063	0.095
2	1169	1017	99	112	752	417	0.82	0.261	0.193
3	1245	1770	52	97	519	725	0.89	0.036	0.071
4	1333	1746	71	132	615	716	0.97	0.022	0.055
5	1405	1882	49	96	634	770	0.97	0.010	0.037
6	1253	1606	57	99	597	658	0.96	0.019	0.052
7	1197	1401	67	102	620	576	0.97	0.022	**0.056**
8	1217	1485	44	71	610	609	0.93	0.022	0.052
mean ± SD	1237 ± 101	1513 ± 298	64 ± 18	100 ± 17	616 ± 65	620 ± 122	0.93 ± 0.05	0.057 ± 0.084	0.076 ± 0.050

	Gaussian fit					Goodness of fit		
subject	TTP [ms]	FWHM [ms]	PH [%]	AUC [%·s]	Δt [ms]	R^2	SSE	RMSE
1	1217	1137	70	83	211	0.91	0.058	0.091
2	1273	997	99	105	413	0.89	0.157	0.150
3	1361	1257	55	72	250	0.74	0.084	0.109
4	1465	1233	77	100	398	0.85	0.105	0.123
5	1514	1225	54	70	460	0.83	0.061	0.093
6	1397	1201	61	76	350	0.85	0.068	0.099
7	1349	1129	69	82	370	0.88	0.077	0.105
8	1365	1209	46	58	309	0.91	0.029	0.060
mean ± SD	1368 ± 95	1174 ± 84	67 ± 17	81 ± 16	345 ± 84	0.86 ± 0.06	0.080 ± 0.038	0.104 ± 0.026

	Bi-component fit									Goodness of fit		
subject	TTP [ms]	FWHM [ms]	PH [%]	AUC [%·s]	ratio (PH_G/ PH_Γ)	TTP_G [ms]	TTP_Γ[ms]	Δt_G [ms]	Δt_Γ [ms]	R^2	SSE	RMSE
1	1153	1145	70	114	3.48	1117	2418	242	419	**0.98**	**0.014**	**0.060**
2	1181	869	101	157	2.32	1157	2478	543	580	**0.97**	**0.049**	**0.111**
3	1205	1417	54	112	1.54	1125	2603	282	603	**0.98**	**0.006**	**0.038**
4	1361	1381	75	145	1.61	1305	2568	446	500	**0.98**	**0.011**	**0.052**
5	1401	1594	52	104	1.28	1341	2498	546	610	**0.98**	**0.005**	**0.037**
6	1281	1269	60	112	1.79	1229	2498	397	500	**0.98**	**0.008**	**0.046**
7	1229	1033	70	119	1.98	1197	2442	499	542	**0.98**	**0.015**	0.061
8	1285	1237	46	81	2.58	1233	2623	317	623	**0.98**	**0.006**	**0.035**
mean ± SD	1262 ± 87	1243 ± 229	66 ± 17	118 ± 24	2.07 ± 0.71	1213 ± 81	2516 ± 74	409 ± 119	547 ± 70	0.98 ± 0.01	0.014 ± 0.015	0.055 ± 0.025

Supporting Table S2. Average metrics for eight ROIs for Left Upper (LU), Left Middle (LM), Low, Right Upper (RU), Right Middle (RM), LU and Low meninges Lymphatic Vessels (mLV), Superior Sagittal Sinus (SSS).

	units	Left Middle (LM)	Low	Low mLV	Left Upper (LU)	Left Upper (LU) mLV	Right Middle (RM)	Right Upper (RU)	SSS
SIR_{MAX}	%	122.5 ± 80.7	70.8 ± 41.9	84.9 ± 42.5	84.5 ± 50.2	104.9 ± 66.4	26.7 ± 11.9	24.4 ± 12.8	89.6 ± 53.3
Δt_G	ms	266.1 ± 161.2	369.1 ± 58.7	326.0 ± 109.5	361.0 ± 119.2	229.8 ± 129.4	333.3 ± 155.2	407.0 ± 145.2	402.7 ± 103.5
Δt_Γ	ms	319.4 ± 196.7	591.6 ± 129.8	445.4 ± 208.8	409.5 ± 157.4	263.3 ± 177.1	482.2 ± 15.1	515.5 ± 241.3	440.0 ± 100.5
AUC	%·s	204.7 ± 149.4	86.2 ± 66.7	107.6 ± 67.5	131.5 ± 90.7	161.6 ± 112.9	30.5 ± 15.1	35.4 ± 17.9	112.8 ± 63.0
TTP	ms	1223.2 ± 154.1	1315.6 ± 115.8	1201.2 ± 230.3	1261.8 ± 115.2	1188.7 ± 156.0	1468.6 ± 233.9	1619.6 ± 633.8	1373.4 ± 319.1
TTP_G	ms	1177.2 ± 137.9	1267.0 ± 69.8	1150.2 ± 191.0	1230.2 ± 113.1	1162.7 ± 154.9	1115.6 ± 191.8	1606.6 ± 714.6	1129.1 ± 202.6
TTP_Γ	ms	2319.7 ± 195.5	2591.4 ± 129.7	2445.8 ± 208.7	2409.8 ± 157.1	2264.3 ± 176.7	2289.5 ± 197.1	2516.2 ± 241.0	2440.2 ± 100.3
FWHM	ms	1651.2 ± 591.1	1245.2 ± 540.3	1215.2 ± 558.7	1147.6 ± 133.4	1139.1 ± 125.1	955.5 ± 129.2	1453.0 ± 632.7	1278.8 ± 700.6
PH	%	124.7 ± 93.2	61.3 ± 43.2	79.4 ± 40.0	84.2 ± 54.5	106.2 ± 73.9	24.6 ± 11.2	23.3 ± 12.1	85.3 ± 51.6
PH_G	%	99.3 ± 76.4	53.6 ± 37.0	68.7 ± 33.0	68.4 ± 43.9	88.1 ± 61.6	22.0 ± 10.1	19.1 ± 10.0	75.1 ± 46.2
PH_Γ	%	33.6 ± 26.3	11.4 ± 10.6	15.1 ± 14.6	21.4 ± 15.7	24.2 ± 18.8	4.0 ± 3.8	6.2 ± 3.5	15.8 ± 8.2
AUC	%·s	204.7 ± 149.4	86.2 ± 66.7	107.6 ± 67.5	131.5 ± 90.7	161.6 ± 112.9	30.5 ± 15.1	35.4 ± 17.9	112.8 ± 63.0
ratio (PH_G/PH_Γ)		1.6 ± 1.1	1.9 ± 1.4	4.7 ± 0.9	2.9 ± 1.0	4.1 ± 0.6	2.6 ± 2.1	2.8 ± 1.5	4.1 ± 2.1

SIR_{MAX} – max measured raw signal increase ratio

Δt_G, Δt_Γ – arrival delay for Gaussian and Γ-variate components respectively

AUC – area under the curve,

TTP, TTP_G, TTP_Γ – time-to-peak for bi-component fit, Gaussian and Γ-variate components

FWHM – full-width at half-maximum

PH, PH_G, PH_Γ – peak height for bi-component fit, Gaussian and Γ-variate components
